# Defining work-focused cognitive behavioural therapy (W-CBT) and whether it is effective at facilitating return to work for people experiencing mental health conditions: A systematic review and narrative synthesis

**DOI:** 10.1177/20551029231217840

**Published:** 2023-11-24

**Authors:** Dylan Slater, Anthony Venning, Lynda Matthews, Ross Iles, Paula Redpath

**Affiliations:** 1 Discipline of Behavioural Health, College of Medicine & Public Health, 1065 Flinders University, Adelaide, SA, Australia; 2 Faculty of Medicine and Health, 445681 University of Sydney, Sydney, NSW, Australia; 3 Healthy Working Lives Research Group, School of Public Health and Preventive Medicine, 2541 Monash University, Clayton, VIC, Australia

**Keywords:** cognitive behaviour therapy, mental health, return to work, systematic review, work focused cognitive behaviour therapy

## Abstract

It is unclear what constitutes Work Focused Cognitive Behaviour Therapy (W-CBT). This review sought to define W-CBT and ascertain its effectiveness at facilitating return to work (RTW) for people experiencing mental health conditions. A systematic review and narrative synthesis were undertaken. Five databases were searched (Medline, ProQuest, PsychInfo, Scopus, and Web of Science). English publications with an intervention combining CBT with RTW were selected. Quality checklists from the Joanna Briggs Institute were applied. Searching yielded 16,863 results. 23 moderate-to-high quality studies from 25 articles were included (13 experimentally designed studies, 3 pilots/case studies and 7 reviews). Results indicated W-CBT is effective at facilitating RTW for mild-to-moderate mental health conditions. For a program to be labelled W-CBT it is recommended it is (1) a stand-alone intervention; (2) delivered with an understanding RTW is the goal; and, (3) the CBT components are always framed by matters, subjects and contexts related to work.

## Introduction

The social and health benefits good work can offer people, their families, and society is well documented (Harrex and Chase, 2022). Employment can be beneficial for people’s health (Hoefsmit et al., 2012) and contribute to greater quality of life (De Boer et al., 2015), as it can facilitate social integration and contributes to social identity (Mikkelsen and Rosholm, 2018). On the other hand, not all work is beneficial for people’s health (Taouk et al., 2020). For example, work-related stressors are associated with an increased likelihood of sick leave from work (Hultén et al., 2023; Hulten et al., 2022). Nevertheless, it is widely accepted that good work is generally good for health and wellbeing (Harrex and Chase, 2022) and long-term leave from work can be detrimental to businesses, employers, individuals, families, and society. The majority of industrialised nations acknowledge long-term sick leave as an escalating public health issue, with significant personal, and socioeconomic consequences (Henderson et al., 2005; CHRC, 2007; OECD OfECaD, 2010). From a global perspective, the Organisation for Economic Co-operation and Development (OECD) reported that, in 2014, workers in OECD countries lost an average of two weeks work due to sick leave (OECD, 2016). In the same year, the average self-reported number of days lost due to illness, for Australian workers, was 7.3 days (OECD, 2014). However, these statistics fail to properly account for long-term sick leave, as no formal, national recording system in Australia currently exists.

Research has shown there is an inverse relationship between the duration of an individuals’ sick leave and the likelihood they will return to work (RTW) (Vogel et al., 2017). For example, a study showed that being sick-listed for six months or longer resulted in an 80% chance of remaining absent from work for 5 years (Waddell, 2006 in Vogel et al., 2017). Furthermore, once an individual is on disability benefits, they tend to move onto other benefits, or retire, rather than RTW (OECD of ECaD, 2010). As such, there is a need to ensure appropriate supports and interventions are implemented to aid in reducing duration of sick leave and facilitate RTW. Australian guidelines for the treatment of workplace related mental health conditions recommend general practitioners (1) refer to existing high-quality interventions while considering work-related factors, and (2) consider a referral for Work-Focused Cognitive Behaviour Therapy (W-CBT) when the primary condition is musculoskeletal (Mazza et al., 2019).

Therapies that fall under the Cognitive Behaviour Therapy (CBT) umbrella have a large evidence base supporting their use for treating a range of mental health conditions, including anxiety (Hofmann and Smits, 2008; Rector et al., 2014), depression (Angelakis et al., 2022), panic (Pompoli et al., 2016), social anxiety (Mayo-Wilson et al., 2014), Generalised Anxiety Disorder (GAD) (Covin et al., 2007; Kishita and Laidlaw, 2017), Obsessive Compulsive Disorder (OCD) (Reid et al., 2021), and specific phobia (Hofmann and Smits, 2008). However, a systematic review of RTW interventions found Traditional Cognitive Behaviour Therapy (T-CBT) was not effective at increasing RTW rates for mental health conditions, whereas W-CBT was, as well as reducing the amount of sick leave taken (Cullen et al., 2018). Accordingly, Cullen et al. (2018) recommended W-CBT be implemented to facilitate RTW and reduce costs over T-CBT. However, exactly what W-CBT is, how and by whom it is implemented, and how it differs from T-CBT is unclear. The Australian guidelines for the treatment of workplace related mental health conditions do not offer a definition of W-CBT (Mazza et al., 2019) and Cullen et al. (2018) briefly suggested W-CBT is CBT focused on work relevant solutions. They cite adjunctive occupational therapy running parallel to T-CBT (Schene et al., 2007; Hees et al., 2013), T-CBT with an integrated work-focused module delivered by psychotherapists (Lagerveld et al., 2012) and guided self-help with workplace guidance delivered by labour experts (Blonk et al., 2006) as examples of W-CBT. Occupational therapy running parallel to CBT differs from Lagerveld et al.'s (2012) original conception of W-CBT where it was emphasized the work focused intervention was not a separate course of treatment. In their supplementary material, they stated W-CBT should simultaneously address a person’s presenting problems while facilitating return to work.

Considering CBT is an umbrella term and there appears to be a large difference in the modalities across studies attributing RTW success to W-CBT, this review aims to define W-CBT, describe its component parts, and evaluate whether it is effective at facilitating RTW.

## Methods

### Search strategy

A systematic review of five databases was undertaken. Drawing on the Preferred Reporting Items for Systematic Reviews and Meta Analyses (PRISMA) guidelines (Moher et al., 2009), a narrative synthesis was completed to group together and describe the components of W-CBT. An expert librarian assisted to define the search terms (refer to Supplemental Appendix A) and as recommended (Godin et al., 2015), six experts were contacted to inform a search of the grey literature (refer to Supplemental Appendix B). Subsequently, an advanced Google search was conducted with the first 200 results screened (Bramer et al., 2017). Finally, a scoping search of industry and government websites was conducted for grey literature. In keeping with a previous review of work connected interventions for people with psychological injuries (Iles et al., 2020), the scoping search included: The Department of Veteran’s Affairs (DVA), Comcare (Australia’s national authority for work health and safety), New South Wales’s State Insurance Regulatory Authority (SIRA), Black Dog institute, Super friend, Phoenix Australia, Employers Mutual Limited, Beyond Blue, Safework (NSW and SA), Worksafe (VIC, QLD, NT, ACT, TAS), and The National Institute for Health and Care Excellence (NICE).

### Inclusion criteria

Studies were included if they met the following criteria:

• Participants were aged 18 years or older and on full or partial sick leave, or unemployed and trying to return to paid employment.
• Participants were experiencing mental health conditions that could affect the working aged population, such as depression, anxieties and adjustment conditions. The mental health conditions were not secondary to another condition. For example, pain related, or musculoskeletal disorders were not included (even if secondary mental health concerns were present).
• Studies were written in English.
• Interventions consisted of a combination of CBT and a return-to-work intervention delivered by one practitioner, not a combination of two or more practitioners. This is in-keeping with Lagerveld et al.'s (2012) original conception of W-CBT.
• Studies had a RTW outcome measuring changes to employment status, sick leave, or work hours.

### Study screening and selection

One author (DS) independently screened the titles and abstracts of all articles. Where abstracts met the inclusion criteria, the full text was reviewed by two authors independently (DS and AV). Reference lists of identified publications were also checked for additional relevant studies. Discrepancies were discussed leading to agreement upon final studies for inclusion.

### Critical appraisal of included studies

The quality of included studies’ methodology were critically appraised using checklists from the Joanna Briggs Institute (higher scores indicating higher quality). A combination of two authors (DS & AV, and DS & LM) independently appraised each study. When ratings differed, consensus was achieved via discussion. In line with other reviews utilising checklists from the Joanna Briggs Institute, studies with a score of 50% or less were excluded from the review to ensure a consistent level of quality (Venning et al., 2021). No authors were contacted as part of the quality appraisal, as it was considered this may have negatively impacted further planned qualitative research regarding W-CBT.

### Data extraction and synthesis

Data was extracted by one reviewer (DS). General study characteristics and impact on mental health symptoms was extracted from all selected studies. Data included the study location, sample size, mental health conditions included, exclusion criteria, impact of W-CBT on mental health and whether the conditions were work-related or not. At times, a secondary reference was located (e.g., an intervention protocol). On some occasions, assumptions were made; for example, if a study included a sample of participants from an employer, it was deemed reasonable the RTW was to the same employer. Furthermore, none of the studies reported they delivered intervention via telehealth, so it was assumed sessions were conducted face-to-face. Unless it was clearly defined, it was also assumed RTW was subsequent from a first period of sick leave.

For the included reviews, data extracted included type of review, objective, study types, participants, and the main findings. Three steps of narrative synthesis were followed: (1) develop a preliminary synthesis, (2) explore relationships in the data, and (3) assess the robustness of the synthesised product (Popay et al., 2006). To develop a preliminary synthesis, the data was (a) organised into groups relating to study design, and (b) tabulated.

## Results

### Study search and inclusion

**Figure 1.**
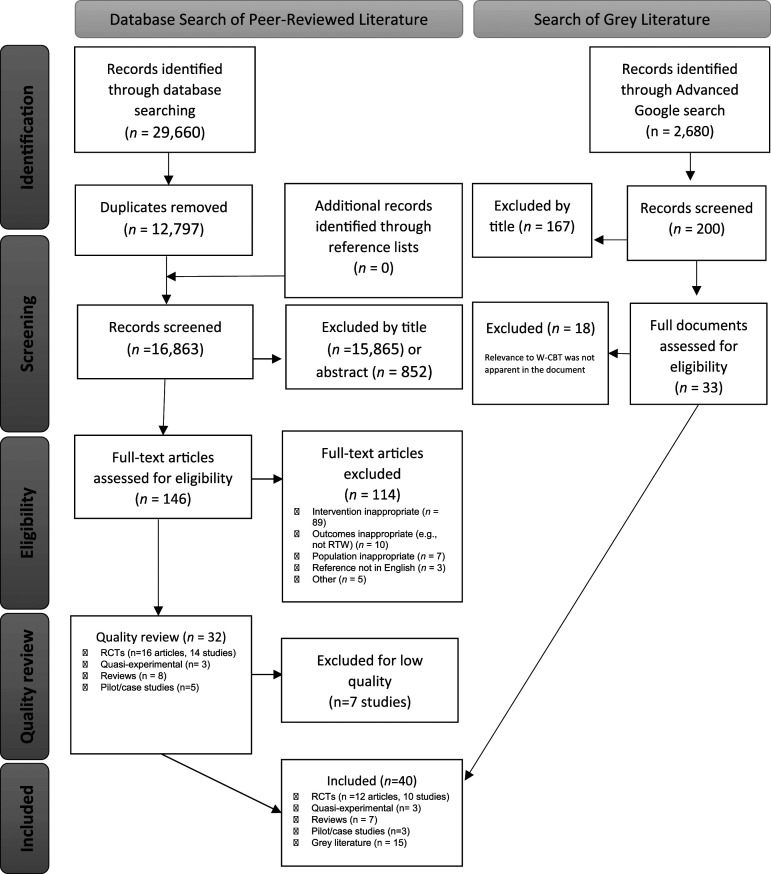
PRISMA flow-chart of study search and selection.

Database searching yielded 29,660 records (Figure 1). Following the removal of duplicates, 16,863 were screened by title with 15,865 excluded. Following screening by abstract, 146 articles were assessed for eligibility via the full text, with 32 screened for quality. These comprised of 14 Randomised Controlled Trials (RCTs), eight review papers, three quasi-experimental designs, and five other types of papers (pilot or case studies). Following quality assessment, 16 studies (comprising 18 articles) and seven reviews were included.

For the grey literature, an advanced Google search occurred on 30 April 2022, alongside a scoping search of the websites listed earlier. The search returned 2680 results (Figure 1). The first 200 were screened and 167 were excluded by title. After review of 33 full documents, 15 were included.

### Quality of included studies

**Figure 2.**
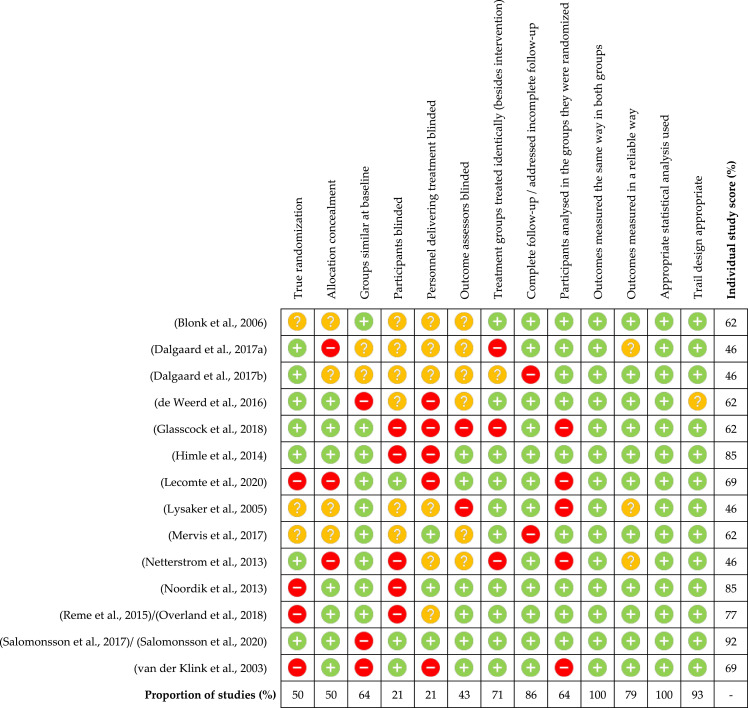
Critical appraisal of randomised controlled trials (RCTs).

Critical appraisal of the RCTs (*n* = 14) is shown in Figure 2. Four were removed for low quality (appraisal scores ≤ 50%) (Dalgaard et al., 2017a, 2017b; Lysaker et al., 2005; Netterstrom et al., 2013). Appraisal scores for the remaining RCTs ranged between 62% and 92% (Blonk et al., 2006; De Weerd et al., 2016; Glasscock et al., 2018; Himle et al., 2014; Lecomte et al., 2020; Mervis et al., 2017; Noordik et al., 2013; Reme et al., 2015; Overland et al., 2018; Salomonsson et al., 2017, 2020; Van der Klink et al., 2003). Trial design, recording of outcome measures, completing following up and statistical analysis were done appropriately in the majority of included RCTs. The biggest limitation across the RCTs concerned blinding. However, it should be acknowledged this is practically difficult for studies regarding psychological interventions.

Following critical appraisal of the quasi-experimental studies (*n* = 3) (refer to Supplemental Appendix C) all studies were included as they had scores greater than or equal to 50% (56%-100%) (Gjengedal et al., 2020; Kroger et al., 2015; Lagerveld et al., 2012). Clear cause and effect, similarities in participants, existence of a control group, consistency in measurements used, and appropriate statistical analysis were done appropriately in most of the included quasi-experimental studies.

Following critical appraisal of the reviews (*n* = 8), one was removed because of low quality (appraisal score ≤ 50%) (Minjoo et al., 2014). Appraisal scores for the remaining reviews ranged between 64% and 100% (refer to Supplemental Appendix D) (Cullen et al., 2018; Axén et al., 2020; Dewa et al., 2015; Noordik et al., 2010; Stergiopoulos et al., 2011; Torchalla and Strehlau, 2018; Gaillard et al., 2020). Appropriate inclusion criteria, appropriate search strategy, appropriate sources, appropriate methods of combining data, and recommendations for policy was identified in most of the included reviews. Likelihood of publication bias was the most common limitation identified.

Following critical appraisal of the pilot or case review studies (*n* = 5), two were removed from the systematic review due to low quality (appraisal scores ≤50%) (Ito et al., 2019; Davis et al., 2005). Appraisal scores for the remaining pilot or case studies ranged between 80 and 100% (refer to Supplemental Appendix E) (Hellerstein et al., 2015; Kukla et al., 2019; Winter et al., 2020).

### General study characteristics of all studies (*n* = 16)

General characteristics of all studies are shown in Table 1. Studies were conducted in The Netherlands (6), USA (4), Germany (2), Sweden (1), Norway (1), Denmark (1) and Canada (1). Total sample sizes ranged from 16 to 1193 participants (M = 177). In sum, there were 2825 subjects across all studies. Ten studies (63%) did not specify whether the mental health condition was work-related or not (Blonk et al., 2006; De Weerd et al., 2016; Noordik et al., 2013; Salomonsson et al., 2017; Gjengedal et al., 2020; Kroger et al., 2015; Lagerveld et al., 2012; Kukla et al., 2019; Hellerstein et al., 2015; Winter et al., 2015). One study (6%) included work-stress as an inclusion criterion (Glasscock et al., 2018) and in five (31%) it was inferred the mental health condition did not have to be work related to be included (Himle et al., 2014; Lecomte et al., 2020; Mervis et al., 2017; Overland et al., 2018; Van der Klink et al., 2003). Improvements in mental health following W-CBT were reported in 15 of the 16 (94%) studies. In the other study mental health data was not reported upon (Kukla et al., 2019). In the 13 studies with a comparison group, none reported symptoms of mental health were significantly worse after W-CBT compared to comparisons. The most common condition treated was adjustment disorder (*n* = 9), followed by depressive conditions (*n* = 6) and anxiety (*n* = 6). Stress and burnout were part of the inclusion criteria in five studies. The most common reason for exclusion was substance abuse/dependence (*n* = 11), followed by psychosis (*n* = 6).

**Table 1.** General study information, mental health conditions treated, exclusion criteria and reported impact of symptoms in all studies (*n* = 16).

Study	Quality score (%)	Country	N	Mental health conditions included	Work related condition	Exclusion criteria	Impact on symptoms
(Blonk et al., 2006)	62	NLD	122	AD, job stress and burnout	?	Serious psychiatric disorder (MDD, anxiety, addictive disorders, PTSD)	A reduction in psychological symptoms was present in each group of the study with no significant differences between groups
(De Weerd et al., 2016)	62	NLD	60	Sick listed with CMDs	?	Psychotic disorders, acute suicidality, substance abuse/addictive disorders, severe personality disorders, or mental health conditions with no evidence base for CBT	Improvement in mental health was noted for participants receiving W-CBT and W-CBT with a CDM. No significant differences between groups was found
(Glasscock et al., 2018)	62	DNK	137	Diagnosis of AD, or stress reaction	Yes	PTSD; mild depression; substance abuse; sick leave > 4 months; resigned from job before intervention	A significant improvement in mental health and perceived stress was recorded in the intervention group with moderate effect size in the first 4 months of intervention
(Himle et al., 2014)	85	USA	58	SAD	No*	Substance dependence, opiate use, cocaine use, psychosis, manic symptoms, suicidal/homicidal ideation with imminent risk	Significantly greater reductions in depression, general anxiety, social anxiety, and functional impairment was recorded in the group receiving W-CBT compared to the comparison group
(Lecomte et al., 2020)	69	CAN	164	Severe mental health disorder (schizophrenia, BP, MDD). Substance abuse/cognitive deficits or social deficits were not an exclusion criterion	No*	Inability to communicate in English or French; known organic disorder; IQ below 70; previously received CBT.	The group who were treated with W-CBT experienced a significant decrease in their negative symptoms over time, compared to the control group
(Mervis et al., 2017)	62	USA	64	Schizophrenia-spectrum disorder	No*	Evidence of substances abuse	Those in the intervention group reported significantly fewer defeatist beliefs and greater motivation to participate in community work compared to those assigned to the support group condition
(Noordik et al., 2013)	85	NLD	160	CMDs (stress-related disorders; anxiety, depression and AD)	?	Somatic disorder, not able to speak Dutch and post-traumatic stress disorder	In all groups distress, depression, anxiety, and somatization scores decreased significantly over time. There were no statistically significant between group differences
(Reme et al., 2015)/(Overland et al., 2018)	77	NOR	1193	CMDs (anxiety and depression) on or at risk of sick leave. On benefits; had to express motivation to work/stay at work	No*	Other problem than mental health was causing problems with work (low motivation to work); severe psychiatric disorders. High suicide risk, pregnancy, ongoing substance abuse	The intervention group reported significant improvements in mental health at 12-month follow up. These were more favourable than the control group
(Salomonsson et al., 2017)/(Salomonsson et al., 2020)	92	SWE	211	MDD, social phobia, GAD, PTSD, panic disorder (w/out agoraphobia), OCD, specific phobia, insomnia, AD, exhaustion disorder	?	Psychosis, BP, dementia, self-harm, eating disorder, substance abuse	Large improvements from pre-to-post treatment were recorded in all groups, which were sustained at 1 year follow up
(Van der Klink et al., 2003)	69	NLD	192	First sickness because of AD.	No*	Excluded if the checklist pointed to other confounding mental health condition. If treatment/guidance had previously been sought for AD. If there was a physical co-morbidity that could affect absenteeism. Pregnancy, recent child delivery (<6 months), if employment would terminate within 6 months	There was no difference between groups in the decrease in symptoms
**Quasi-experimental designs**							
(Gjengedal et al., 2020)	56	NLD	182	Mild to moderate symptoms of depression, anxiety or AD.	?	Severe mental health disorder (personality disorder, BP, psychosis, high suicide risk, substance abuse. Unemployed, studying on parental leave, receiving disability/unemployment benefits or working without sick leave benefits	Effect sizes for self-efficacy, depression and anxiety were large in the intervention group and significantly lower in the control group
(Kroger et al., 2015)	100	DEU	26	Met criteria for MDD	?	Mental retardation, dementia, BP, schizophrenia, anorexia. Alcohol dependence	Moderate to large effect size was recorded for all treatment groups in this study
(Lagerveld et al., 2012)	89	NLD	168	AD, undifferentiated somatoform disorder, anxiety disorder or a mood disorder	?	Severe disorder: MDD, PTSD.	A significant decrease in mental health conditions was equally present in all treatment groups
**Case studies/pilots**							
(Kukla et al., 2019)	90	USA	52	Diagnosis of a severe mental illness	?	Significant cognitive deficit, major medical condition inhibiting participation in CBT or work	Effect on symptoms was not measured
(Hellerstein et al., 2015)	80	USA	16	Chronic MDD or DD and unemployed	?	Cognitive or psychotic disorder, bipolar, eating disorder, severe BPD, alcohol or drug dependence (except nicotine), unstable medical condition, psychotropic medications except antidepressants	Behavioural avoidance and global functioning improved significantly
(Winter et al., 2020)	100	DEU	20	Diagnosis of MDD	?	Substance abuse, eating disorder, bipolar, physical disorder, acute suicidality	There was a statistically significant reduction in depressive symptoms from pre-test to post-test

*Inferred from information in the study. *Note:* DEU = Germany; NLD = Netherlands; USA = United States of America; NOR = Norway; SWE = Sweden; CAN = Canada; DNK = Denmark; AD = Adjustment Disorder; BP = Bipolar depression; BPD = Borderline Personality Disorder; CDM = Convergence Dialogue Meeting; CMDs = Common Mental Health Disorders; DD = Double Depression; GAD = Generalised Anxiety Disorder; MDD = Major Depressive Disorder; PTSD = Post-Traumatic-Stress-Disorder; OCD = Obsessive Compulsive Disorder; SAD = Social Anxiety Disorder.

### Overall return to work results in experimentally designed studies (*n* = 13)

Table 2 presents details including who facilitated intervention programs, the intervention program duration, session durations, session frequency, delivery format, the RTW outcome measures, the type of RTW, and the results on RTW. It should be noted, none of the studies reported they delivered intervention via telehealth. It can therefore be assumed all intervention sessions were conducted in person.

**Table 2.** Interventions, intervention characteristics and return-to-work outcomes for experimentally designed studies (*n* = 13).

Study	Interventions	Provider	Duration (sessions)	Session length (min) (mins)	Frequency (per week)	Format	Number of strategies	Outcome	Return to work measure	Results on return to work
**RCTS**										
(Blonk et al., 2006)	CI (*n* = 40)	Labour experts	6	60	2	1:1	6	Return to same employer*	Days until partial and full return to work	RTW was significantly quicker in the combined intervention group for both partial and full RTW. For full RTW the difference was approx. 200 days
	CBT (*n* = 42)	Psychotherapists	11	45	2	1:1	N/A			
	Control (n=40)	General Practioner (GP)	“breif”	2	4-monthly	1:1	N/A			
(De Weerd et al., 2016)	W-CBT (*n* = 29)	Registered or in-training psychologist	6	?	1.5	1:1	10	Return to same employer*	Days until partial and full return to work	The time before partial and full return to work did not differ significantly between groups
	W-CBT with CDM (*n* = 31)	Registered or in-training psychologist	6	?	1.5	1:1	11			
(Glasscock et al., 2018)	W-CBT (*n* = 57)	Psychologists	6	60	<1	1:1	8	Return to same employer	Full time resumption of work for four consecutive weeks following sick leave	There was no significant difference between groups regarding sick leave before full resumption of work for four consecutive weeks
	CAU/no treatment (*n* = 80)	?	?	?	?	?	?			
(Himle et al., 2014)	W-CBT + VSAU (*n* = 29)	Vocational service employees	8	120	2	G	5	New employer	Hours of work	No significant differences in hours of work were found between groups
	VSAU (*n* = 29)	Vocational service employees	N/A	N/A	N/A	N/A	N/A			
(Lecomte et al., 2020)	CBT-SE (*n* = 79)	Graduate level therapists	8	60	2	G	6	New employer	Hours of work	The intervention group worked significantly more hours and significantly more obtained competitive paid employment at follow up compared to the control group
	SE (85)	Vocational service employees	N/A	N/A	N/A	N/A	N/A			
(Mervis et al., 2017)	IVIP (*n* = 29)	Graduate level therapists	8	45	1	G	6	New employer	Obtain supported employment	Significantly more in the intervention group obtained supported employment compared to the control group
	Support group (*n* = 35)	Graduate level therapists	?	45	1	G	N/A			
(Noordik et al., 2013)	RTW-E (*n* = 75)	Occupational physician (OP)	3.9 sessions on average	?	?	1:1	4	Same employer*	Days until partial and full return to work	Workers receiving RTW-E returned to full work significantly later than the control group. There was no significant difference for partial return to work
	CAU (*n* = 85)	Occupational physician (OP)	3.4 sessions on average	?	?	1:1	As per guideline-based care			
(Reme et al., 2015)/(Overland et al., 2018)	AWaC (*n* = 630)	Therapists (clinical psychologist) and employment specialists	15	?	?	1:1	4	Mix of same employer and new employer	Work participation (working and reduced benefits compared to baseline)	Significantly more participants in the AWaC group increased or maintained work participation at 1 year compared to the control group. At 18 months follow up the difference remained significant. Over the 10-months to 46-months longer term follow up, the intervention group had more months without receiving benefits, but the differences were not statistically significant
	CAU (*n* = 563)	General Practitioner (GP), health care professionals and employment specialists	Unrestricted	?	?	?	?			
(Salomonsson et al., 2017)/(Salomonsson et al., 2020)	CI (*n* = 80)	Licensed psychologists	25	?	ATN	1:1	8	Same employer*	Days of sick leave	There was no significant differences between groups regarding amount of sick leave at 1 year follow up
	RTW-I (*n* = 67)	Licensed psychologists	10	?	<1	1:1	N/A			
	CBT (*n* = 64)	Licensed psychologists	20	?	ATN	1:1	N/A			
(Van der Klink et al., 2003)	Intervention (*n* = 109)	Occupational physician (OP)	5	90	<1	1:1	5	Same employer*	Percentage of subjects returning to work partially and amount of sickness leave at 12 months	At 3 months significantly more participants RTW in the intervention group. Sick leave was significantly shorter in the intervention group compared to the control group
	CAU (*n* = 83)	Occupational physician (OP)	5	?	?	1:1	?			
**Quasi-experimental designs**										
(Gjengedal et al., 2020)	W-MCBT/CBT (*n* = 87)	Clinical psychologists, psychiatrists, or psychiatric nurses	10	?	?	1:1	5	Same employer*	Full return to work	Significantly more subjects returned to full work in the intervention group compared to the control group
	Waitlist control (*n* = 95)	General practitioner (GP)	?	?	?	?	?			
(Kroger et al., 2015)	W-CBT (*n* = 13)	Post graduate CBT students	25	?	1	1:1	8	Same employer*	Partial and full return to work	At follow up significantly more subjects were fully working in the W-CBT group compared to the control. There were no between group differences for partial return to work
	CBT-AU (*n* = 13)	Post graduate CBT students	25	?	1	1:1	7			
(Lagerveld et al., 2012)	W-CBT (*n* = 89)	Psychotherapists	12	?	<1	1:1	11	Same employer*	Partial and full return to work	Subjects receiving W-CBT returned to work significantly faster both partially and fully compared to the control group. For full return to work the median difference between groups was 65 days
	CBT (*n* = 79)	Psychotherapists	12	?	<1	1:1	N/A			

*Note:* *inferred; CBT = Cognitive Behavioural Therapy; W-CBT = Work focused Cognitive Behavioural Therapy; W-CBT with CDM = Work focused Cognitive Behavioural Therapy with a convergence dialogue meeting; VSAU = Vocational services as usual; SE = supported employment; RTW = Return to work; RTW-E = exposure based return to work; IVIP = Indianapolis vocational intervention program; CAU = care as usual; AWaC = At work and coping; CI = combined intervention; RTW-I = return to work intervention; W-MCBT = work focused meta-cognitive behavioural therapy; CBT-AU = Cognitive Behavioural Treatment as usual; ATN = According to need.

Overall, eight studies (62%) reported W-CBT to be more effective than comparisons for facilitating RTW (Blonk et al., 2006; Gjengedal et al., 2020; Kroger et al., 2015; Lagerveld et al., 2012; Lecomte et al., 2020; Mervis et al., 2017; Overland et al., 2018; Van der Klink et al., 2003). Three (23%) found that W-CBT was equal to comparisons (Glasscock et al., 2018; Himle et al., 2014; Salomonsson et al., 2017) and in one study (7%) W-CBT was compared to W-CBT with a workplace meeting (De Weerd et al., 2016) which resulted in no significant differences. One study (7%) found the W-CBT group returned to work significantly later that the comparison (Noordik et al., 2013). In the studies where W-CBT was found to be more efficacious than the comparison at facilitating RTW, these included a total of 1076 participants receiving W-CBT. In the studies where W-CBT was not found to be more efficacious than the comparison conditions, these included a total of 241 participants receiving W-CBT.

In the experimental studies where the quality score was greater than 75% (*n* = 6), three found W-CBT to be more effective than a comparison at facilitating RTW (Reme et al., 2015; Lagerveld et al., 2012; Kroger et al., 2015), two reported no significant differences between groups (Salomonsson et al., 2020; Himle et al., 2014), and one found those receiving W-CBT returned to work significantly later than the comparison conditions (Noordik et al., 2013).

Of the RCTs, three out of the four studies reported W-CBT was not effective at facilitating RTW compared to comparison conditions (Salomonsson et al., 2017; Himle et al., 2014; Noordik et al., 2013). One study reported W-CBT was significantly more effective at facilitating return to work at 12 to 18 months follow up compared to care as usual (Reme et al., 2015). Of note, this study exposed 630 participants to W-CBT compared to 184 participants in the others combined. Only one RCT compared W-CBT to T-CBT, which resulted in no statistical differences in the amount of sick leave between participants (Salomonsson et al., 2017, 2020).

All quasi-experimental studies (Lagerveld et al., 2012; Kroger et al., 2015; Gjengedal et al., 2020) reported W-CBT to be significantly more effective at facilitating RTW than the comparison group. The total number of participants who were exposed to W-CBT in these three studies was 189 with two comparing W-CBT with T-CBT (Lagerveld et al., 2012; Kroger et al., 2015).

In the studies where it was inferred the RTW goal was a return to an existing employer (Blonk et al., 2006; De Weerd et al., 2016; Gjengedal et al., 2020; Glasscock et al., 2018; Kroger et al., 2015; Lagerveld et al., 2012; Noordik et al., 2013; Salomonsson et al., 2017; Van der Klink et al., 2003), five out of nine (56%) reported W-CBT was more efficacious than comparisons at facilitating RTW (Blonk et al., 2006; Gjengedal et al., 2020; Kroger et al., 2015; Lagerveld et al., 2012; Van der Klink et al., 2003). One (11%) compared W-CBT to W-CBT with a workplace meeting (De Weerd et al., 2016). In the three studies where the RTW goal was with a new employer (Himle et al., 2014; Lecomte et al., 2020; Mervis et al., 2017), two (67%) reported W-CBT was more effective at facilitating RTW than comparisons (Lecomte et al., 2020; Mervis et al., 2017).

The longest W-CBT program was 25 sessions and the shortest was a mean duration of 3.9 sessions. The mean program duration of all experimentally designed studies was 10.2 sessions, and the median duration was eight. Seven studies did not report the duration of intervention sessions (De Weerd et al., 2016; Noordik et al., 2013; Reme et al., 2015; Overland et al., 2018; Salomonsson et al., 2017, 2020; Gjengedal et al., 2020; Lagerveld et al., 2012; Kroger et al., 2015), three reported they lasted 1 hour (Blonk et al., 2006; Glasscock et al., 2018; Lecomte et al., 2020), one 90 min (Van der Klink et al., 2003), the shortest session length was 45 min (Mervis et al., 2017), and the longest 120 min (Himle et al., 2014). In three studies, it was unclear how frequently sessions were provided (Gjengedal et al., 2020; Noordik et al., 2013; Overland et al., 2018), three studies provided sessions twice a week (Blonk et al., 2006; Himle et al., 2014; Lecomte et al., 2020), two weekly (Kroger et al., 2015; Mervis et al., 2017), one provided three sessions every two weeks (De Weerd et al., 2016) one provided four-to-five sessions over 6 weeks (Van der Klink et al., 2003), one provided six sessions in four months (Glasscock et al., 2018) one provided 11.1 over 5.5 months (Lagerveld et al., 2012), and one provided sessions that were flexible to needs (Salomonsson et al., 2017).

Ten studies (77%) provided a 1:1 intervention (Blonk et al., 2006; De Weerd et al., 2016; Gjengedal et al., 2020; Kroger et al., 2015; Lagerveld et al., 2012; Noordik et al., 2013; Salomonsson et al., 2017; Glasscock et al., 2018; Overland et al., 2018; Van der Klink et al., 2003) and three (23%) provided interventions in groups (Himle et al., 2014; Lecomte et al., 2020; Mervis et al., 2017). Of the ten studies that delivered W-CBT in a 1:1 format, six (60%) reported W-CBT was more effective than the comparison at facilitating RTW (Blonk et al., 2006; Overland et al., 2018; Van der Klink et al., 2003; Gjengedal et al., 2020; Kroger et al., 2015; Lagerveld et al., 2012). One compared W-CBT to W-CBT with a workplace meeting (De Weerd et al., 2016). Of the three studies that delivered W-CBT via a group format, two (67%) reported it to be more efficacious than the comparison groups at facilitating RTW (Lecomte et al., 2020; Mervis et al., 2017).

Eight studies reported six or more strategies used in the intervention protocol (Blonk et al., 2006; De Weerd et al., 2016; Kroger et al., 2015; Lagerveld et al., 2012; Salomonsson et al., 2017; Glasscock et al., 2018; Lecomte et al., 2020; Mervis et al., 2017). Of these eight studies, five (62%) reported W-CBT was more efficacious at facilitating RTW than comparisons (Blonk et al., 2006; Lecomte et al., 2020; Mervis et al., 2017; Lagerveld et al., 2012; Kroger et al., 2015). There were five studies who used five or less strategies in their intervention protocol (Gjengedal et al., 2020; Noordik et al., 2013; Himle et al., 2014; Overland et al., 2018; Van der Klink et al., 2003). Of these five studies, three (60%) reported W-CBT to be more efficacious at facilitating RTW than comparisons (Gjengedal et al., 2020; Overland et al., 2018; Van der Klink et al., 2003).

Nine studies reported the duration of intervention was ten or less sessions (Blonk et al., 2006; De Weerd et al., 2016; Gjengedal et al., 2020; Glasscock et al., 2018; Himle et al., 2014; Lecomte et al., 2020; Mervis et al., 2017; Van der Klink et al., 2003; Noordik et al., 2013). Of these, five (55%) reported W-CBT effective at facilitating RTW (Blonk et al., 2006; Gjengedal et al., 2020; Lecomte et al., 2020; Mervis et al., 2017; Van der Klink et al., 2003). One study compared W-CBT to W-CBT with a workplace meeting (De Weerd et al., 2016). In three of the four studies where the duration was eleven sessions or more, W-CBT was reported to be more effective than comparisons at facilitating RTW (Kroger et al., 2015; Lagerveld et al., 2012; Reme et al., 2015). For two of the studies, follow-up data was collected at 12-months (Lagerveld et al., 2012; Kroger et al., 2015) and one of the studies it was collected at 18 months (Reme et al., 2015) and 46 months (Overland et al., 2018).

### W-CBT strategies used in all studies (*n* = 16)

**Figure 3.**
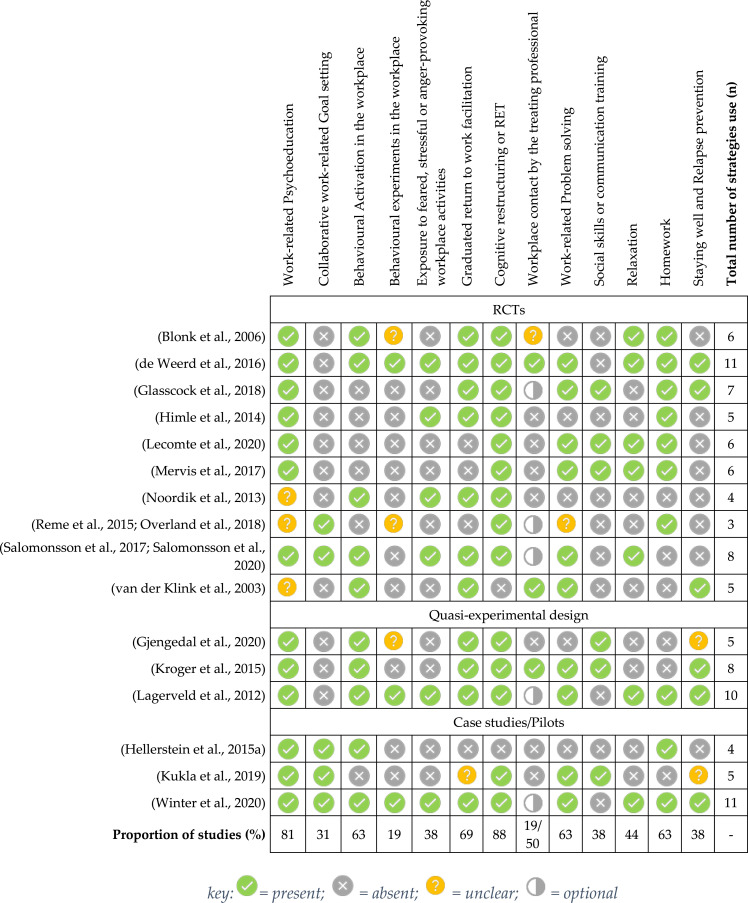
Number and types of W-CBT strategies used across all studies (*n* = 16).

Figure 3 shows the number and type of CBT strategies used in W-CBT interventions across the sample of included studies (*n* = 16). All studies utilised multiple W-CBT strategies in their intervention. The number of strategies used ranged from three to 11 (Lagerveld et al., 2012; Winter et al., 2020; De Weerd et al., 2016).

Work related psychoeducation was included in 13 (81%) studies and in the other three it was unclear whether this was provided. The work-focused aspect was either psychoeducation on work stress (Blonk et al., 2006; Lecomte et al., 2020; Glasscock et al., 2018) or work-related psychoeducation (Hellerstein et al., 2015; Himle et al., 2014; Gjengedal et al., 2020; Kroger et al., 2015; Kukla et al., 2019; Lagerveld et al., 2012; Overland et al., 2018). In five studies (31%), collaborative work-related goal setting was explicitly stated as part of their W-CBT intervention. Only one of the studies that included goal setting reported W-CBT was effective at facilitating RTW (20%) (Reme et al., 2015). Behavioural activation at work was included in ten studies (63%). This was a reduction in work demand and subsequent increase, sometimes via the use of a hierarchy (De Weerd et al., 2016; Lagerveld et al., 2012; Winter et al., 2020). Hierarchies generally facilitated graded activity from structured and simple tasks to unstructured and advanced ones. The term “behavioural experiment” was explicitly stated in three studies (18%) (De Weerd et al., 2016; Lagerveld et al., 2012; Winter et al., 2020), and three studies (Blonk et al., 2006; Gjengedal et al., 2020; Overland et al., 2018) indicated the workplace could be used as a testing ground. It was unclear if formal behavioural experiments were conducted in these three studies. Exposure to fear, anxiety, stress or anger provoking work activities was supported in six studies (38%), four of which used a hierarchy (De Weerd et al., 2016; Lagerveld et al., 2012; Winter et al., 2020; Noordik et al., 2013). Facilitating Graded Return to Work (GRTW) was apparent in 11 studies (69%). Increasing hours spent at work as the intervention progressed was evidence of this component. Cognitive techniques were included in 14 W-CBT protocols (88%). This was described as Rational Emotive Therapy (Blonk et al., 2006), ABCs of CBT, (Lecomte et al., 2020; Overland et al., 2018) or the “four a model” (Davis et al., 2005; Kukla et al., 2019). One study provided meta-cognitive intervention (Gjengedal et al., 2020). Workplace contact was a feature of W-CBT in eight studies (50%), being optional in five (Glasscock et al., 2018; Lagerveld et al., 2012; Overland et al., 2018; Salomonsson et al., 2017; Winter et al., 2020). Contact was in the form of meetings, correspondence or via telephone. One study received written reports from the workplace (Kroger et al., 2015) and in another, intervention sessions could take place at the workplace (Blonk et al., 2006). However, it was unclear whether contact with workplace personnel occurred concurrently to sessions held at the workplace. Work-place problem solving happened in ten studies (63%). This was described as overcoming barriers or obstacles (Lecomte et al., 2020; Davis et al., 2005; Kroger et al., 2015; Kukla et al., 2019) problems and aims (Glasscock et al., 2018), or described in the context of a work task analysis (Lagerveld et al., 2012; Winter et al., 2020; De Weerd et al., 2016). Six studies (38%) reported they provided work-related social or communications skills training. This included negotiation of work accommodations (Lecomte et al., 2020), discussing disclosures at work (Lecomte et al., 2020; Gjengedal et al., 2020), communicating effectively at work (Kukla et al., 2019), accepting and responding to feedback (Kukla et al., 2019) or communicating assertively (Lecomte et al., 2020). Relaxation was a component of the intervention in seven studies (44%). Ten studies (63%) reported homework was given and six (38%) explicitly stated there was a relapse prevention or staying well plan. In three studies, it was unclear whether relapse prevention was implemented. This was because the description was ambiguous (e.g., a success plan).

### Reviews (*n* = 7)

General study information and main findings of the included reviews are shown in Table 3. All included reviews were systematic and one included meta-analysis. All reviews broadly evaluated the effectiveness of interventions for mental health conditions on RTW, apart from one, which was concerned with the economic impact of RTW interventions that had a work-focused element (Gaillard et al., 2020). The total number of included studies ranged from 6 to 36 (111 in total). However, in the review that contained 36 studies, only ten included interventions for mental health (Cullen et al., 2018). Four studies included in this review (Blonk et al., 2006; Kroger et al., 2015; Lagerveld et al., 2012; Van der Klink et al., 2003) were included in four other reviews (Axén et al., 2020; Cullen et al., 2018; Dewa et al., 2015; Gaillard et al., 2020). Across all the included reviews there were 43 individual studies, indicating a 9% overlap of studies included in this review. Ten of the 39 studies had been previously ruled out and 29 were ruled out upon subsequent review.

**Table 3.** General study information, intervention characteristics, and findings from included reviews (*n* = 7).

Reference	Type	Objective of review	Number of studies	Study types included	Findings
(Axén et al., 2020)	Systematic review and narrative synthesis	Synthesise occupational health service interventions targeting prevention or reduction of CMD among employees	33 (*n* = 12,121)	RCTs, meta-analysis, observational study, mixed methods	W-CBT and problem-solving interventions shortened time to initial RTW for employees on sick leave for CMD compared with treatment-as-usual. However, the effect on return to full-time work and improvement of CMD symptoms was inconsistent
(Cullen et al., 2018)	Systematic review	Synthesise the evidence of the effectiveness of work-placed based interventions	36 (*n* = ?)	RCTs, controlled trials, cohort studies	W-CBT was associated with a strong level of evidence for reducing time lost and costs associated with the lost time from work
(Dewa et al., 2015)	Systematic review	Assess the evidence for RTW interventions that incorporate problem-solving skills for workers with sickness absence related to MH disorders	6 (*n* = 975)	RCTs	Limited evidence interventions that contain work-related problem-solving skills are effective for facilitating RTW outcomes
(Gaillard et al., 2020)	Systematic review	Analyse effectiveness of interventions intended to improve employee MH, prevent CMDs, or promote RTW after an absence because of CMDs	11 (*n* = ?)	RCTS and one quasi-experimental design	There is strong evidence of positive economic results for RTW interventions for society and employers
(Noordik et al., 2010)	Systematic review and meta-analysis	Effectiveness of exposure in vivo interventions on RTW	11 articles with 7 studies (*n* = 691)	RCTS, controlled trials	Interventions with exposure in vivo can improve work functioning for those with OCD and PTSD. More research is required to see how this can translate most appropriately to RTW practices
(Stergiopoulos et al., 2011)	Systematic review	Examine the evidence of the effectiveness of interventions adapted for work-related PTSD	7 (*n* = 212)	RCT, quasi-experimental designs	Work-related interventions show promise as effective strategies for promoting RTW for those who acquire PTSD in the workplace. More research is needed
(Torchalla and Strehlau, 2018)	Systematic review	Summarise evidence base for interventions targeting work-related PTSD	11 (*n* = 532)	RCTs, quasi-experimental and cohort studies	Only after traditional treatment for PTSD fails to facilitate RTW, more intensive and multi-disciplinary intervention with a work-specific exposure elements should be included

*Note:* CMDs = Common Mental Health Conditions; RCTs = Randomised controlled trials; MH = Mental Health; OCD = Obsessive Compulsive Disorder; RTW = Return to Work; PTSD = Post-Traumatic Stress Disorder; W-CBT = Work focused CBT.

Of the eight reviews, one (Cullen et al., 2018) concluded that there was strong evidence to support W-CBT’s efficacy in facilitating RTW. One reported it was effective at facilitating first (partial) return to work (Axén et al., 2020). One review reported work-related interventions show promise as an effective strategy for promoting RTW for those who acquire PTSD in the workplace (Stergiopoulos et al., 2011). One review reported interventions with exposure in vivo can improve work functioning for those with OCD and PTSD (Noordik et al., 2010) however another reported intervention with a work-specific exposure element should be implemented if traditional treatment for PTSD fails (Torchalla and Strehlau, 2018). Two reviews found limited evidence indicating work-related interventions are effective for facilitating RTW (Dewa et al., 2015; Gaillard et al., 2020).

### Grey literature (*n* = 15)

Information about the grey literature (15) is shown in Table 4. The majority (9) were forms of evidence synthesis with recommendations on the management of mental health issues and RTW. Of these, six (67%) supported implementation of W-CBT.

**Table 4.** Document information and recommendations for RTW for mental health conditions from included grey literature.

Author	Year	Title	Document aim	Relevance recommendations	Source
Victoria and transport accident commision	2012	Clinical framework for the delivery of health services	Provide principle-based guidance for the treatment of people on sick leave due to any work-related injury or illness	Measure and demonstrate the effectiveness of any intervention (e.g., utilise reliable and valid measurements to track variables over time); deliver education about nature of injury/illness and biopsychosocial models of health; facilitate functional improvements in all areas of life; enable self-management via strategies (e.g. goal setting, behavioural activation, pacing); utilise SMART (specific, measurable, achievable, relevant, timed) goals focused on enhancing function, participation, and RTW; use evidence-based interventions	Comcare
Comcare	2014	Working for recovery: Suitable employment for return to work following psychological injury	Provide structured guidance for managers and rehabilitation case managers to assist people on sick leave due to work-related psychological problems return to work	Intervene early; perform a comprehensive assessment; establish capacity for work; establish suitable duties; identify strengths; facilitate behavioural activation in all areas of life; encourage problem solving to overcome barriers and constraints; create a RTW plan with appropriate RTW goals; facilitate graduated RTW; address unhelpful thoughts and beliefs about pain or injury; encourage adaptive coping strategies; increase self-efficacy; promote durable RTW outcomes	Comcare
Worksafe Tasmania	2018	Managing workplace injuries in Tasmania: A handbook for primary treating medical practitioners	A handbook for medical practitioners involved in managing people on sick leave due to any work-related injury or illness	Measure and demonstrate the effectiveness of treatment; identify psychosocial obstacles such as maladaptive behaviours or unhelpful beliefs, dysfunctional social relationships, or systematic/policy issues and implement strategies to address them; foster hope for RTW and recovery; provide education on remaining active and the issues with avoidant coping mechanisms; adopt a strengths based approach; provide health education when appropriate; use SMART goals focused on function and RTW; promote adoption of a healthy lifestyle; encourage pacing and graded exposure to activities when appropriate; promote evidence based health care	Worksafe Tasmania
British association for supported employment (BASE)	2015	Symptoms of depression and their effects on employment	Research seeking to understand the impacts of depression on employment and examine effectiveness of interventions that may help people experiencing depression to remain in work or find employment	There is moderate quality evidence work focused psychological approaches for improving employment related outcomes for those experiencing depression are effective	BASE
SuperFriend	2018	Taking action: A best practice framework for the management of psychological claims in the australian workers’ compensation sector	A framework to provide insurers and claims managers to better support people on sick leave due to work-related psychological problems	Intervene early (<3-month from first sick day); provide client centered services; use problem solving strategies; identify and communicate timeframes for RTW and recovery; encourage early and graduated RTW; monitor and supervise RTW; use easy to understand language, especially in written form; conduct a biopsychosocial risk assessment; monitor progress against agreed RTW goals; use an explicit work-focus; foster hope for RTW; utilise teletherapy when appropriate	Safe Work Australia
NSW state insurance regulatory authority (SIRA)	2019	Best practice for vocational rehabilitation programs	A rapid review aiming to identify best practice for vocational rehabilitation programs, components of rehabilitation programs and the needs of target groups being addressed in vocational rehabilitation programs	There is strong evidence that incorporating multiple components, specifically incorporating health care, service coordination and workplace/employer components consistently resulted in improved work-related outcomes	SIRA
NICE	2019	Workplace health: Long-term sickness absence and capability to work: Evidence review for facilitating the return to work of employees on long-term sickness absence and reducing risk of recurrence	Evidence review for facilitating the return to work of employees on long-term sickness absence and reducing the risk of recurrence	There were no effects found supporting workplace focused interventions for facilitating RTW in people experiencing mental health problems	NICE
Office of the assistant secretary for planning and evaluation (ASPE)	2017	Work-focused interventions for depression: Final report	Summary of current knowledge regarding the adoption and benefits of work-based depression programs	In comparison to EAP and health promotion programs work-based interventions are more efficacious. It was reported effective work-based depression programs consisted of assessment, psychoeducation, care management, counseling, medication and had a work-focus	ASPE
Institute for safety, compensation and recovery research (ISCRR)	2015	Interventions to improve return to work outcomes in individuals with mental health conditions	A review that evaluated which types of interventions are effective at improving RTW outcomes in people with AD and depression	For people experiencing AD, partial and full RTW was shorter following problem-solving therapy-based interventions and W-CBT compared to CAU (which excluded problem solving aspects). In workers receiving CBT and CAU, time until RTW was similar. This suggests CBT does not improve RTW outcomes in workers experiencing AD. The authors also identified moderate quality evidence that the addition of a work-directed intervention to a clinical intervention reduced the number of sick days in workers diagnosed with depression compared to clinical intervention alone	ISCRR
Institute for work & health and SafetyNet centre for occupational health & safety (IWH&SNCOHS)	2017	Managing depression in the workplace: A systematic review contextualized for Manitoba	A report providing a synthesis of the relevant research-based evidence on managing depression for the adult working population of Manitoba	There is a moderate level of evidence that W-CBT is effective at facilitating RTW after a bout of sick leave because of depression	IWH & SNCOHS
Pathways project	?	Participation to healthy workplaces and inclusive strategies in the work sector	Scoping paper on the available evidence on the effectiveness of existing integration and re-integration into work strategies for persons with chronic conditions and mental health conditions in Europe	Studies implementing W-CBT in the Netherlands and Germany reported positive change in employed people with CMDs either on sick leave or at risk of it	Pathways project
Ontario society of occupational therapists (OSOT)	2020	Occupational therapy in mental health and RTW	Provides guidance to help people understand the role of an occupational therapist assist an individual with a mental health issue return to work	W-CBT focuses on work challenges, specifically difficulties with tasks and duties while at work, in addition to the work environment. Strong evidence supports use of programs with a W-CBT approach to improve RTW outcomes	OSOT
Safework NSW	2017	Review of evidence of interventions to reduce mental ill-health in the workplace	A literature review providing a high-level summary of the strength of evidence for interventions designed to reduce workplace mental ill-health, and issues arising when appraising and implementing these interventions	There are consistent findings provided by several high quality experimental studies work focused psychological therapy supports recovery and RTW.	Safework NSW
SIRA	2020	Work-connected interventions for people with mental health conditions: A literature review	Summary of existing evidence to describe the effectiveness of work-connected interventions for people with psychological injury	The peer review literature regarding work-connected interventions was mixed. The authors found a significant amount of research and knowledge in work-related mental health programs as well as a range of workplace-focused resources, which did not convey an understanding of the long-term effectiveness of them	SIRA
The royal australian college of general practitioners (RACGP)	2019	Clinical guideline for the diagnosis and management of work-related mental health conditions in general practice	Document to provide australian general practitioners (GPs) with the best available evidence to guide their diagnosis and management of patients with work-related mental health conditions	Recommends practitioners refer to existing high-quality guidelines for the management of mental health conditions, while considering work-related factors. In those with a work-related mental health condition secondary to a musculoskeletal injury, W-CBT may be considered	RACGP

*Note:* CMD = common mental health disorder; AD = Adjustment Disorder; CBT = Cognitive Behavioural Therapy; GP = General Practitioner; RTW = Return to work; W-CBT = Work focused CBT; CAU = Care as usual.

The remaining six documents outlined guidelines for RTW practice, which were considered relevant to the implementation of W-CBT. Comcare (2014), SuperFriend (2018) and Worksafe Tasmania (2018) have provided RTW guidelines for individuals sick listed because of work-related mental health conditions. They recommended a response or intervention be: (1) early; (2) use problem solving strategies; (3) include planning, goal setting, and progress reviews; (4) target and improve work capacities via identification of suitable duties; and (5) encourage the individual to engage in RTW.

## Discussion

The current systematic review sought to define Work-Focused Cognitive Behaviour Therapy (W-CBT) and whether it is effective for facilitating return to work (RTW) for people experiencing mental health conditions. The review synthesized information from moderate to high quality randomised controlled trials (RCTs) (10), quasi-experimental studies (3), case or pilot studies (3), and review papers (7), in addition to reviewing 15 documents from the grey literature. Most of the research came from northern or central Europe and concerned people experiencing common mood, anxiety, or adjustment conditions. Results indicated that W-CBT contained thirteen work focused cognitive behavioural strategies and could be provided in both low and high intensity formats by a range of diversified professionals within a stepped care framework. Results indicated W-CBT was shown to be more effective at facilitating RTW (or reducing sick leave) with the same or a new employer than services as usual (which included the provision of Traditional Cognitive Behaviour Therapy (T-CBT)) for people experiencing mild-to-moderate mental health conditions. Moreover, W-CBT improved mental health comparable to care or services as usual, including T-CBT.

Results suggest W-CBT should be a term exclusive to a standalone intervention where CBT is combined with a RTW intervention. This dictates that CBT is delivered with an understanding return to work is the goal of intervention and thus strategies and techniques are framed by matters, subjects and contexts related to work.

A clear differentiation between what is W-CBT and what is not (e.g. T-CBT or T-CBT plus occupational rehabilitation) could prevent T-CBT interventions with a RTW agenda item or T-CBT with parallel occupational rehabilitation being labelled W-CBT. It will also aid the development of clearer guidelines and recommendations for practitioners and researchers when implementing or researching RTW interventions.

### Practical recommendations

Given the current findings, the authors suggest that the primary components of W-CBT are work related goal setting, work related psychoeducation, work-related behavioural activation with a gradual return to work plan, work related problem solving, work-related cognitive therapy and homework.

This recommendation is consistent with previous research which indicated multicomponent interventions with a work focus, including gradual RTW, were amongst the most effective interventions seeking to facilitate RTW for those experiencing mental health conditions (Mikkelsen and Michael, 2018; Cullen et al., 2018; Nieuwenhuijsen et al., 2020). Given W-CBT as an intervention is focused on facilitating RTW, it was surprising only 31% of the studies identified explicitly stated work related goal setting was part of the protocol. Furthermore, in the studies that reported W-CBT was more effective at facilitating RTW than a comparison group, only one study reported work-related goal setting was included (Reme et al., 2015). Because W-CBT should have an explicit and shared goal of facilitating RTW, excluding work related goal setting from W-CBT interventions is potentially counter-intuitive and unprincipled. This is because setting goals based on function or return to work is a core pillar of evidence based allied health practice in Australia (Comcare, 2014) and is an effective behaviour change technique (Epton et al., 2017). Furthermore, goal consensus is probably an effective element in the formation of therapeutic relationships, which contribute significantly to psychotherapeutic outcomes (Norcross and Wampold, 2011). Additionally, if an intervention goal is kept covert (either intentionally or unintentionally) it may enhance the risk of ethical issues in service delivery.

Cognitive restructuring was a part of the intervention protocol in 88% of the experimental studies that reported W-CBT was effective at facilitating RTW. Cognitive restructuring addresses unhelpful attitudes and beliefs (Chand et al., 2021) and can be utilized alongside work-related psychoeducation to address those that are unhelpful for RTW. However, one cannot simply think themselves back to work or back to better health, therefore the main active components associated with effective W-CBT interventions were found to be behavioural activation, graduated return to work planning, problem solving and homework, which is why they are recommended for any future iterations of W-CBT programs. Behavioural activation combined with a graduated RTW plan may be the definitive component that separates W-CBT from T-CBT. This is because graduated RTW plans are not a standard component of T-CBT protocols. Furthermore, RTW plans have the potential to provide the scaffolding where matters, agenda items or tasks are framed with a work focus. It is also worth noting previous research suggests workers are more likely to RTW if they have a return to work plan in place (Sheehan et al., 2018).

These findings support an argument that appropriately scaffolded experiential learning in the workplace contributes to enhanced RTW outcomes. It is possible that workplace-based experiential learning enhances work-related self-efficacy, which is associated with accelerated RTW (Brenninkmeijer et al., 2019; Lagerveld et al., 2017; Volker et al., 2015). It is also feasible that through the process of change, positive outcomes are achieved, self-regulation and self-management can be facilitated (alongside skills and resources to maintain them) contributing to successful behaviour change maintenance (Kwasnicka et al., 2016).

However, tailoring W-CBT in the most effective way requires further research to ascertain which combinations of intervention fit best for individuals and their unique conditions and needs. For example, it is unclear whether the inclusion of relaxation techniques in W-CBT contribute to enhanced RTW outcomes. Furthermore, when only considering the studies that facilitated RTW towards the same employer, the participants in these studies were predominantly experiencing mild-to-moderate symptoms of mental health conditions. The mean depression level of participants in the study with the largest number of participants (Reme et al., 2015) was in the normal range and the mean anxiety severity was measured in a range that was classified as “borderline normal”. In four out of the other five experimental studies supporting W-CBT, the average scores on psychometric testing generally fell in the mild-to-moderate range (Blonk et al., 2006; Van der Klink et al., 2003; Gjengedal et al., 2020; Lagerveld et al., 2012). This suggests more research is required to ascertain W-CBT’s efficacy in populations experiencing higher severity mental health conditions.

Within the United Kingdom’s (UK) National Health Service (NHS), Psychological Wellbeing Workers (PWPs) provide low intensity interventions or guided self-help (GSH) for people experiencing mild-to-moderate conditions within a stepped care framework. Considering previous research generally supports stepped care (Stiles et al., 2019; Ho et al., 2016; Firth et al., 2014), it could be appropriate for W-CBT to be delivered within a GSH/low intensity step for mild-to-moderate conditions. Complex mental health conditions and comorbidities could be supported by higher intensity W-CBT interventions. This is also in keeping with previous research into psychosocial return to work interventions, which recommended low intensity interventions as a practical and logical starting point (Venning et al., 2021).

### Limitations

Several limitations are worth noting. There was a lack of clarity around whether participants returning to the same employer were returning to pre-condition duties, permanent modified ones, or alternate duties. The lack of details around the critical demands of the workplace in the context of a mental health condition limits the ability to forecast how long and intensive a W-CBT intervention could or should be. For example, a person with a mild condition returning to a workplace with complicated and unstructured workplace demands may require higher intensity support than a person with a complex condition returning to a workplace with simple and structured demands. Therefore, future research should incorporate whether a person has a work related condition or not, explicitly state whether the RTW is with the same employer or a different employer and record the critical demands of the workplace where the person is returning to. This will provide richer insights to inform future practice.

Studies included in this review were mainly from Europe, so the findings lack generalisability across geopolitical and societal contexts. Therefore, the recommendations of this review need to be considered in the context of environmental and contextual factors. The bundling of mental health conditions into one category, which includes both mood and anxiety conditions amongst others also makes it difficult to draw robust conclusions. When delivering T-CBT, different conditions have different treatment protocols, which means future research would be more useful if it clearly differentiated which W-CBT intervention strategies were implemented for each condition.

Finally, findings from this systematic review should be considered within the constraints of some methodological limitations. A meta-analysis of the included studies could not be performed due to considerable heterogeneity in W-CBT interventions, samples used, and the assessment of RTW outcomes across studies. These diverse elements impeded synthesis of the evidence to draw more reliable conclusions. Methodological weaknesses of some included studies (e.g., low participant numbers, no treatment or waitlist control groups and lack of effect size calculations) also made it difficult to evaluate the effectiveness of W-CBT interventions, which dilutes the recommendations outlined in this review. In the six experimental studies that used care or services as usual as a control group, the services participants received was not clearly reported. In four of the studies (Gjengedal et al., 2020; Reme et al., 2015; Lecomte et al., 2020; Glasscock et al., 2018) it was difficult to determine if the control groups accessed an intervention, and if they did, what type and intensity of support they received. Future studies should try to avoid this issue by explicitly specifying CAU activities (Fong et al., 2015). Despite findings indicating W-CBT improves mental health for those experiencing mental health conditions only three studies reported on effect size of improvements (Glasscock et al., 2018; Gjengedal et al., 2020; Kroger et al., 2015). Despite these being a moderate to large effect size, previous research is mixed (Dewa et al., 2015; Nieuwenhuijsen et al., 2020). Therefore, future research that measures and reports on the effect sizes on mental health is also required.

## Conclusion

Results indicate W-CBT is effective at facilitating RTW outcomes for people experiencing mild-to-moderate mental health conditions. It is recommended for a program to be labelled W-CBT it is a stand-alone intervention where CBT is delivered with an understanding RTW is the goal. Thus, W-CBT strategies and techniques are always framed by matters, subjects and contexts related to work. When designing W-CBT programs, the results suggest practitioners should consider the following components first and foremost: work related goal setting, work related psychoeducation, work-related behavioural activation with a gradual return to work plan, work related problem solving, work-related cognitive therapy and homework. A clear definition of W-CBT allows it to be differentiated from other forms of CBT or RTW interventions ensuring it is delivered by design and not just in name.

## Supplemental Material

Defining work-focused cognitive behavioural therapy (W-CBT) and whether it is effective at facilitating return to work for people experiencing mental health conditions: A systematic review and narrative synthesis Click here for additional data file. Supplemental Material for Defining work-focused cognitive behavioural therapy (W-CBT) and whether it is effective at facilitating return to work for mental health disorders: A systematic review and narrative synthesis by Dylan Slater, Anthony Venning, Lynda Matthews, Ross Iles, Paula Redpath in Health Psychology Open

## Data Availability

Data sharing is not applicable to this article as no datasets were generated or analysed during the current study. All relevant information is included in the manuscript.
