# Supplementary material for: Defining work-focused cognitive behavioural therapy (W-CBT) and whether it is effective at facilitating return to work for people experiencing mental health conditions: A systematic review and narrative synthesis
Source: Health Psychol Open. 2023 Nov 24;10(2):20551029231217840. doi: 10.1177/20551029231217840 (PMC10676636; doi:10.1177/20551029231217840)
Supplement: Defining work-focused cognitive behavioural therapy (W-CBT) and whether it is effective at facilitating return to work for people experiencing mental health conditions: A systematic review and narrative synthesis [file sj-pdf-1-hpo-10.1177_20551029231217840.pdf]

Appendix A

**Medline**	**Scopus**	**Web of science**	**Psychinfo**	**Proquest**
exp employment/ or work engagement/ or work/ or reemployment/	N/A	N/A	exp employment/ or work engagement/ or work/ or reemployment/	N/A
(workplace or worker* or (work adj3 (site or location or related)) or job or indust* or vocation* or occupant* or "work reintegration" or "vocational reintegration" or "return to employment" or unemploy* or "back to work" or "back-to-work" or "return to work" or "worker* compensation").ti, ab,kf.	(workplace or worker* or (work W/2 (site or location or related)) or job or indust* or vocation* or occupant* or "work reintegration" or "vocational reintegration" or "return to employment" or unemploy* or "back to work" or "back-to-work" or "return to work" or "worker* compensation" or (sick* W/2 leave) or (sick W/2 list*) or (Sick W/2 absen*) or unemploy* or (medical W/2 leave or absentee* or presenteeism or "sick day" or "illness day" or "disability leave" or "time off")))	(workplace or worker* or (work NEAR/3 (site or location or related)) or job or indust* or vocation* or occupant* or "work reintegration" or "vocational reintegration" or "return to employment" or unemploy* or "back to work" or "back-to-work" or "return to work" or "worker* compensation")	(workplace or worker* or (work NEAR/3 (site or location or related)) or job or indust* or vocation* or occupant* or "work reintegration" or "vocational reintegration" or "return to employment" or unemploy* or "back to work" or "back-to-work" or "return to work" or "worker* compensation")	(workplace or worker* or (work NEAR/3 (site or location or related)) or job or indust* or vocation* or occupant* or "work reintegration" or "vocational reintegration" or "return to employment" or unemploy* or "back to work" or "back-to-work" or "return to work" or "worker* compensation")
absenteeism/ or sick leave/ or return to work/		N/A	absenteeism/ or sick leave/ or return to work/	N/A

((sick* adj3 leave) or (sick adj3 list*) or (Sick adj3 absen*) or unemploy* or (medical adj3 leave)).ti,ab,kf.		((sick* NEAR/3 leave) or (sick NEAR/3 list*) or (Sick NEAR/3 absen*) or unemploy* or (medical NEAR/3 leave))	((sick* NEAR/3 leave) or (sick NEAR/3 list*) or (Sick NEAR/3 absen*) or unemploy* or (medical NEAR/3 leave))	((sick* NEAR/3 leave) or (sick NEAR/3 list*) or (Sick NEAR/3 absen*) or unemploy* or (medical NEAR/3 leave))
(absentee* or presenteeism or "sick day" or "illness day" or "disability leave" or "time off").ti,ab,kf.		(absentee* or presenteeism or "sick day" or "illness day" or "disability leave" or "time off")	(absentee* or presenteeism or "sick day" or "illness day" or "disability leave" or "time off")	(absentee* or presenteeism or "sick day" or "illness day" or "disability leave" or "time off")
or/1-5				
psychotherapy/ or exp cognitive behavior therapy/ or cognitive therapy/ or Counseling/	N/A	N/A	psychotherapy/ or exp cognitive behavior therapy/ or cognitive therapy/ or Counseling/	N/A
(cbt or psychotherap* or (cognitive adj3 (therapy or therapies))).ti,ab,kf.	(cbt or psychotherap* or (cognitive W/2 (therapy or therapies or counselling or "occupational therapy" or "case manage*" or "absence management" or "assertive community treatment" or "management program*" or rehabilitation)))	(cbt or psychotherap* or (cognitive NEAR/3 (therapy or therapies)))	(cbt or psychotherap* or (cognitive NEAR/3 (therapy or therapies)))	(cbt or psychotherap* or (cognitive NEAR/3 (therapy or therapies)))
7 or 8				

(counselling or "occupational therapy" or "case manage*" or "absence management" or "assertive community treatment" or "management program*" or rehabilitation).ti,ab,kf.		(counselling or "occupational therapy" or "case manage*" or "absence management" or "assertive community treatment" or "management program*" or rehabilitation)	(counselling or "occupational therapy" or "case manage*" or "absence management" or "assertive community treatment" or "management program*" or rehabilitation)	(counselling or "occupational therapy" or "case manage*" or "absence management" or "assertive community treatment" or "management program*" or rehabilitation)
case management/ or community mental health services/ or exp rehabilitation/	N/A	N/A	case management/ or community mental health services/ or exp rehabilitation/	N/A
10 or 11				
9 or 12				
("mental health" or "mental illness" or "mental disorder" or stress or psychiatric or anxiety or depression or "mood disorder" or "post-traumatic stress disorder" or Ptsd or "traumatic stress disorder").ti,ab,kf.	("mental health" or "mental illness" or "mental disorder" or stress or psychiatric or anxiety or depression or "mood disorder" or "post-traumatic stress disorder" or Ptsd or "traumatic stress disorder" or (((affective or "acute stress" or stress or adjustment or neurotic or obsessive-compulsive or phob* or panic or "general* anxiety") W/1 disorder*) or GAD or OCD))	("mental health" or "mental illness" or "mental disorder" or stress or psychiatric or anxiety or depression or "mood disorder" or "post-traumatic stress disorder" or Ptsd or "traumatic stress disorder")	("mental health" or "mental illness" or "mental disorder" or stress or psychiatric or anxiety or depression or "mood disorder" or "post-traumatic stress disorder" or Ptsd or "traumatic stress disorder")	("mental health" or "mental illness" or "mental disorder" or stress or psychiatric or anxiety or depression or "mood disorder" or "post-traumatic stress disorder" or Ptsd or "traumatic stress disorder")

exp anxiety disorders/ or exp depressive disorder/	N/A	N/A	exp anxiety disorders/ or exp depressive disorder/	
(((affective or "acute stress" or stress or adjustment or neurotic or obsessive-compulsive or phob* or panic or "general* anxiety") adj2 disorder*) or GAD or OCD).ti,ab,kf.	(((affective or "acute stress" or stress or adjustment or neurotic or obsessive-compulsive or phob* or panic or "general* anxiety") W/1 disorder*) or GAD or OCD)	(((affective or "acute stress" or stress or adjustment or neurotic or obsessive-compulsive or phob* or panic or "general* anxiety") NEAR/2 disorder*) or GAD or OCD)	(((affective or "acute stress" or stress or adjustment or neurotic or obsessive-compulsive or phob* or panic or "general* anxiety") NEAR/2 disorder*) or GAD or OCD)	(((affective or "acute stress" or stress or adjustment or neurotic or obsessive-compulsive or phob* or panic or "general* anxiety") NEAR/2 disorder*) or GAD or OCD)
14 or 15 or 16				
6 and 13 and 17				

Appendix B – Grey Literature Search Terms

- “Work focused cognitive behaviour therapy”
- W-CBT
- “Work focused CBT”
- English
- Region: All
- File Types: PDF

Appendix C – Critical appraisal of quasi-experimental studies

	Clear cause and effect studied	Participants in comparisons were similar	Comparison group(s) received comparable treatment	There was a control group	Multiple measurements of the outcome both pre and post intervention	Follow up was complete	Outcome of participants measured in the same way	Outcomes were measured reliably	Appropriate statistical analysis completed	**Individual study score (%)**
(Gjengedal et al., 2020)	?	+	−	+	−	−	+	+	+	56
(Kroger et al., 2015)	+	+	+	+	+	+	+	+	+	100
(Lagerveld et al., 2012)	+	+	−	+	+	+	+	+	+	89
**Proportion of studies (%)**	75	100	25	100	75	75	100	100	100	-

Appendix D – Critical appraisal of reviews

	Clearly stated research question	Appropriate inclusion criteria	Appropriate search strategy	Appropriate sources and resources utilised	Appropriate critical appraisal criteria	Appraised by two or more reviewers [illegible]	Appropriate data extraction	Appropriate methods to combine data	Likelihood of publication bias assessed	Recommendations for policy/practice supported by data	Appropriate Specific directives for research	**Individual study score (%)**
(Axén et al., 2020)	+	+	+	+	−	−	−	+	−	+	+	64
(Cullen et al., 2018)	+	+	+	+	+	+	+	+	+	+	+	100
(Dewa et al., 2015)	+	+	+	+	+	+	−	N/A	−	N/A	−	67
("Gaillard, 2020)	+	+	+	+	+	+	+	+	−	+	−	82
("Minjoo, 2014)	−	+	?	−	?	?	?	N/A	−	?	+	20
(Noordik et al., 2010)	−	+	+	+	+	+	+	+	+	+	+	91
(Stergiopoulos et al., 2011)	−	+	+	+	+	+	+	+	+	+	+	91
(Torchalla and Strehlau, 2018)	+	+	+	+	+	+	+	+	−	+	+	91
**Proportion of studies (%)**	63	100	88	88	75	75	63	100	38	86	75	-

Appendix E – Critical appraisal of Pilot or case studies

	Clear criteria for case inclusion	Condition measure in a standardized & reliable way	Valid methods used for identification of the condition	There was consecutive inclusion of participants	There was complete inclusion of participants	Clear reporting of demographics	Clear reporting of clinical information	Outcome/follow up was clearly reported	Clear reporting of presenting sites demographical information	Appropriate statistical analysis	**Individual study score (%)**
(Davis et al., 2005)	−	+	+	?	?	−	−	−	+	N/A	34
(Ito et al., 2019)	+	+	?	?	+	−	−	+	−	+	50
(Hellerstein et al., 2015a)	+	+	?	+	−	+	+	+	+	+	80
(Kukla et al., 2019)	?	+	+	+	+	+	+	+	+	+	90
(Winter et al., 2020)	+	+	+	+	+	+	+	+	+	+	100
**Proportion of studies (%)**	60	100	60	60	60	60	60	80	80	100	-
